# Efficacy and safety of catheter ablation for Brugada syndrome: an updated systematic review

**DOI:** 10.1007/s00392-022-02020-3

**Published:** 2022-04-22

**Authors:** Yasuhito Kotake, Sumita Barua, Samia Kazi, Sohaib Virk, Ashwin Bhaskaran, Timothy Campbell, Richard G. Bennett, Saurabh Kumar

**Affiliations:** 1 Department of Cardiology, Westmead Hospital, Westmead, NSW Australia; 2 Westmead Applied Research Centre, University of Sydney, Westmead, NSW Australia

**Keywords:** Brugada syndrome, Catheter ablation, Abnormal substrate, Provocation strategy

## Abstract

**Background:**

Patients with Brugada syndrome (BrS) may experience recurrent ventricular arrhythmias (VAs). Catheter ablation is becoming an emerging paradigm for treatment of BrS.

**Objective:**

To assess the efficacy and safety of catheter ablation in BrS in an updated systematic review.

**Methods:**

We comprehensively searched the databases of Pubmed/Medline, EMBASE, and Cochrane Central Register of Controlled Trials from inception to 11th of August 2021.

**Results:**

Fifty-six studies involving 388 patients were included. A substrate-based strategy was used in 338 cases (87%), and a strategy of targeting premature ventricular complex (PVCs)/ventricular tachycardias (VTs) that triggered ventricular fibrillation (VF) in 47 cases (12%), with combined abnormal electrogram and PVC/VT ablation in 3 cases (1%). Sodium channel blocker was frequently used to augment the arrhythmogenic substrate in 309/388 cases (80%), which included a variety of agents, of which ajmaline was most commonly used. After ablation procedure, the pooled incidence of non-inducibility of VA was 87.1% (95% confidence interval [CI], 73.4–94.3; *I*^2^ = 51%), and acute resolution of type I ECG was seen in 74.5% (95% CI [52.3–88.6]; *I*^2^ = 75%). Over a weighted mean follow up of 28 months, 7.6% (95% CI [2.1–24]; *I*^2^ = 67%) had recurrence of type I ECG either spontaneously or with drug challenge and 17.6% (95% CI [10.2–28.6]; *I*^2^ = 60%) had recurrence of VA.

**Conclusion:**

Catheter ablation appears to be an efficacious strategy for elimination of arrhythmias or substrate associated with BrS. Further study is needed to identify which patients stand to benefit, and optimal provocation protocol for identifying ablation targets.

**Graphical abstract:**

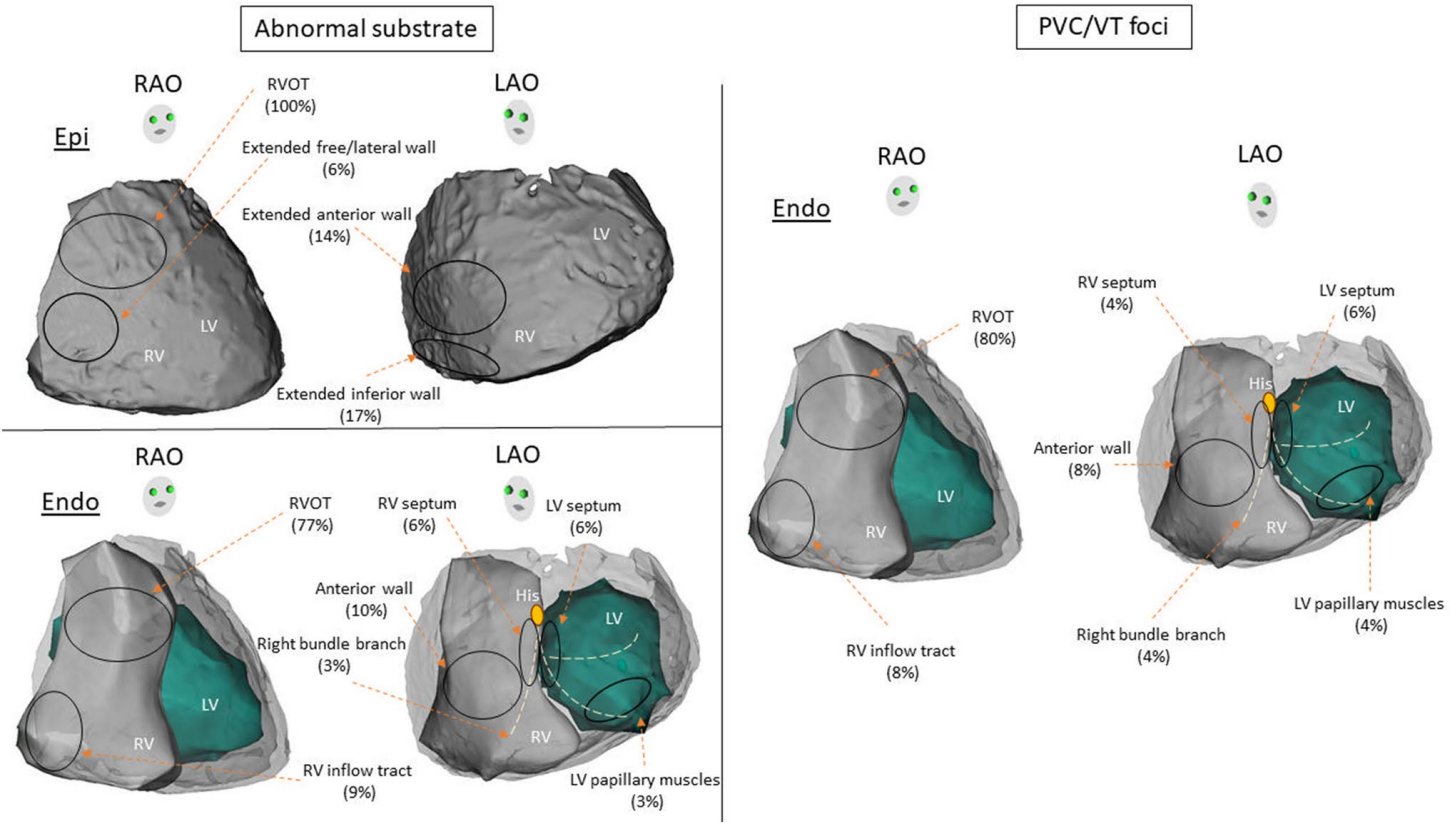

**Supplementary Information:**

The online version contains supplementary material available at DOI: 10.1007/s00392-022-02020-3.

## Introduction

Brugada syndrome (BrS) is an inherited cardiac condition characterized by a coved-shaped ST-segment elevation (Type 1) on 12-lead electrocardiography (ECG) and is associated with risk of sudden cardiac death. Following appropriate risk stratification, insertion of implantable cardioverter defibrillator (ICD) may prevent sudden cardiac death from ventricular arrhythmias (VA) in high-risk individuals [1]. Recurrent lethal VA in this population occurs at an estimate rate of 17% over a follow-up period of ~ 7 years [2]. Furthermore, ~ 2% may experience electrical storm which typically tends to cause recurrent ventricular fibrillation (VF) almost exclusively occurring in those patients with a prior resuscitated cardiac arrest. Recurrent VA can lead to significant morbidity and adverse psychological sequelae from repeated shocks.

Catheter ablation has emerged as an important therapeutic option for BrS. Initial reports targeted premature ventricular complex (PVC) and/or ventricular tachycardia (VT) felt to be responsible for triggering VF, however these triggers were frequently absent during an ablation procedure [3]. Recently, an approach targeting abnormal substrate for BrS has emerged. Abnormal electrograms (EGMs) with low voltage, fractionated or delayed potentials, and areas of slow conduction were identified predominantly on epicardial, but also endocardial surface of BrS patients, serving as putative targets for ablation. The epicardial right ventricular outflow tract (RVOT) notably harboured abnormal EGM [3] with post-mortem studies showing this to correlate with interstitial fibrosis and reduced gap junction expression [4]. In the absence of substrate abnormalities at baseline sinus rhythm, intravenous sodium channel blockers may uncover substrate regions that could be targeted for ablation, sometimes even beyond the epicardial RVOT [5]. Indeed, a substrate-based catheter ablation appears to provide an attractive adjunct to ICD insertion in high-risk patients with BrS. A number of studies have subsequently described catheter ablation for BrS. We conducted an updated systematic review with a pooled analysis to assess the safety and efficacy of catheter ablation as a therapeutic option for patients with BrS, as published in the literature over the past two decades.

## Methods

### Search strategy

This systematic review was registered in PROSPERO (registration number: CRD42021267328). Pubmed/Medline, EMBASE, and Cochrane Central Register of Controlled Trials databases from inception to 11th of August 2021 were systematically searched to identify all relevant studies. The search terms used were “Brugada syndrome” and “Ablation”. No language restriction was applied at the beginning, however only articles in English were included in the final analysis. The reference list of all identified articles was also reviewed for relevant publications fitting the eligibility criteria. No restriction was applied to the publication date or the age of participants in each study. Due to the paucity of published data on catheter ablation of BrS, we included case reports in the present review.

### Definition of Brugada syndrome

BrS was defined as the presence of a type 1 ECG that was present spontaneously or induced by pharmacological provocation with a sodium channel blocker based on updated expert consensus statement [1].

### Inclusion criteria

The inclusion criteria for this review were studies with the following characteristics:

1. Included patients with a diagnosis of BrS [1].
2. Ablation approach (endocardial, epicardial or combined) was clearly defined.
3. Ablation strategy (substrate-based ablation and/or targeting of PVC/VT) was clearly defined.

Study eligibility and data extraction are outlined in online resource.

### Statistical analysis

Continuous variables were expressed as mean ± standard deviation, or median and 25–75% interquartile range (IQR) when data were skewed. Procedural success rates, incidence of procedural complications and recurrence rate were pooled using DerSimonian–Laird random-effects models to take into account the anticipated clinical and methodological diversity between studies. Summary estimates were reported with 95% confidence intervals (CI). The *I*^2^ statistic was used to represent the proportion of variation between the sample estimates. Statistical analysis was conducted with Comprehensive Meta Analysis v3.3 (Biostat Inc, Englewood, NJ, US).

## Results

### Description of included studies

**Fig. 1:**
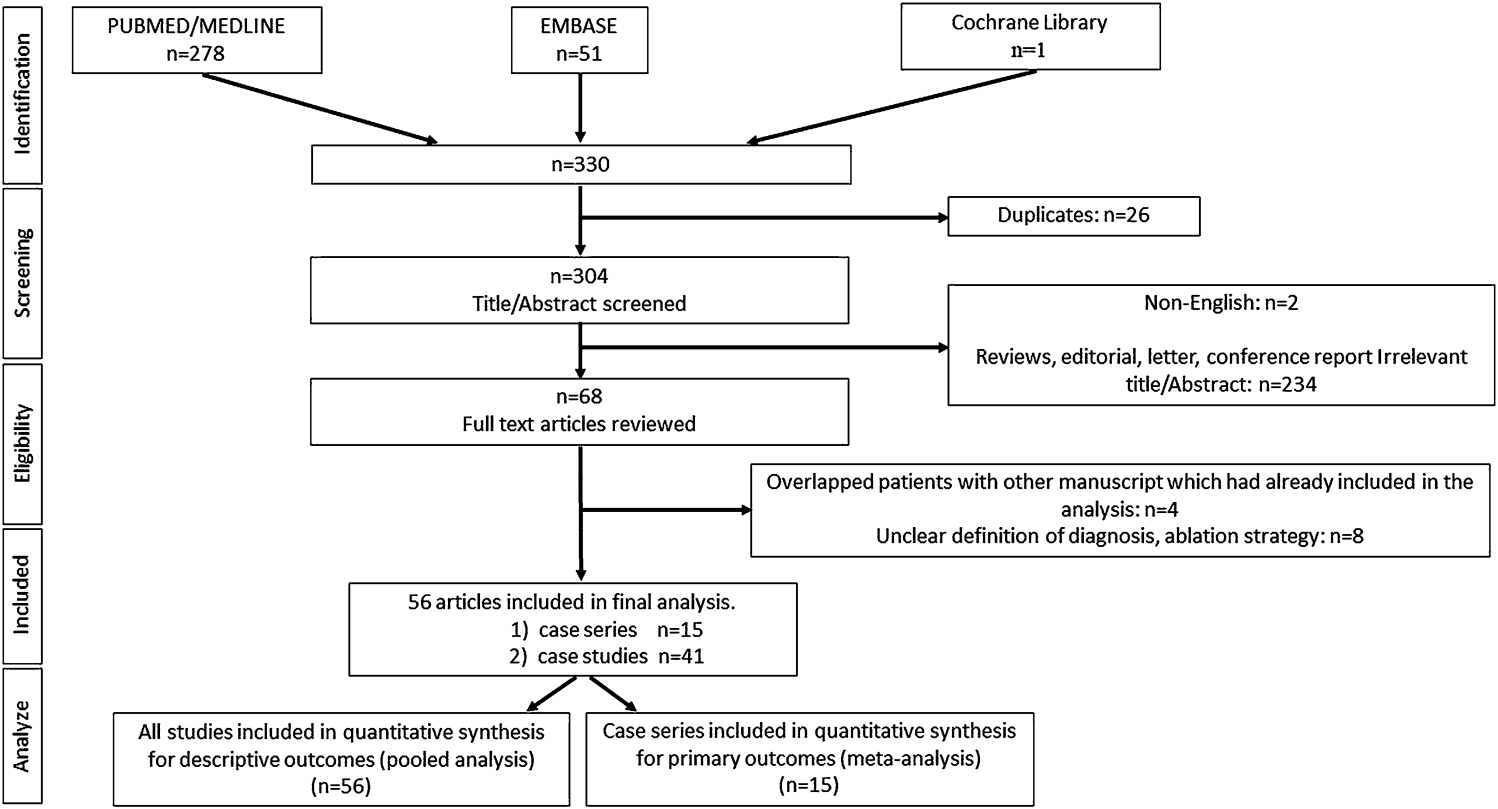
PRISMA (Preferred reporting items for systematic reviews and meta-analyses) flow chart for literature search and study selection

Figure 1 illustrates the flowchart for a literature search and study selection. At the initial systematic searching, 330 potentially relevant articles were identified. After exclusion of 26 duplicate articles, 304 articles underwent title and abstract screening. Of these, 234 articles were excluded at this stage since they did not meet the inclusion criteria described above. Another two articles were excluded since they were written in non-English language. Sixty-eight articles underwent full-text review. Four articles were excluded, as they were derived from the same database that had been used for another manuscript which had already been included in the analysis. Another eight articles were excluded because of unclear description of the ablation strategy. As a result, a total of 56 articles with 388 patients (15 case series [3, 5–18] and 41 case studies [19–59]) were included. Systematic review was performed for descriptive outcomes in all 56 studies and single arm meta-analysis was performed for procedural outcomes of ablation procedures in 15 case series.

### Baseline characteristics

Patient characteristics in all 56 studies with 388 patients are described in Table 1. The majority of patients were male (87%), with a median age of 39 (IQR 34–50) years. Left ventricular (LV) function was reported in 264 patients (68%), all with preserved function. Right ventricular (RV) function was reported on echocardiogram in 241 (62%) patients. In one patient (0.3%), arrhythmogenic right ventricular cardiomyopathy (ARVC) was suspected on echocardiography, however it was unable to be confirmed due to inability to perform a magnetic resonance imaging (MRI) due to the presence of a non-MRI compatible ICD [6]. Cardiac MRI was reported in 158 (41%).

**Table 1:** Patient characteristics

Patient characteristics	*n* = 388
**Gender—male (%)**	337 (87%)
**Age (mean, IQR [year])**	39 (34–50)
**Initial presentation**	***n=342***
Resuscitated cardiac arrest (%)	123 (36%)^a^
Syncope (%)	72 (21%)^a^
Other (nocturnal agonal respiration, palpitation) (%)	147 (43%)^a^
**ICD implantation—yes (%)**	374/386 (97%)^a^
**SCN5A mutation present—yes (%)**	68/257 (26%)^a^
**Previous history of spontaneous sustained VA**	***n=197***
Spontaneous ventricular fibrillation (%)	95 (48%)^a^
Spontaneous sustained monomorphic VT (%)	25 (13%)^a^
Both rhythms (ventricular fibrillation + VT) (%)	6 (3%)^a^
Unspecified sustained VA (%)	71 (36%)^a^
**Induced sustained VA before ablation procedure**	180/227 (79%)^a^
**No documentation of VA prior to ablation procedure**	6/250 (2%)^a^
**Previous VA storm**	43/91 (47%)^a^

^a^ Percentage among patients who were able to collect data properly

*ICD* implantable cardioverter defibrillator, *IQR* interquartile range, *VA* ventricular arrhythmia, *VT* ventricular tachycardia

The patients’ initial presentations were described in 342/388 cases (88%). Among these 342 patients, 123 patients (36%) presenting following resuscitated cardiac arrest, 72 patients (21%) presenting following syncope and 147 patients (43%) presenting with other symptoms including palpitations, atrial arrhythmias and nocturnal agonal respiration.

ICD had been implanted prior to, or at the time of ablation, in 374/386 patients (97%), with 12/386 patients (3%) having declined ICD insertion, and the ICD status of the remaining two cases was not known [15]. Genetic testing for an SCN5A mutation was performed in 257 patients (66%), with 68 patients (26%) testing positive. Subsequent to initial diagnosis, presence of sustained VA prior to ablation procedure was described in 250 patients, of which 197 patients (79%) had documented spontaneous sustained VA, which included VF in 95/197 (48%), sustained monomorphic VT in 25/197 (13%), both rhythms (VT and VF) in 6/197 (3%) and unspecified sustained VA in 71/197 (36%). Forty-seven patients (19%) had not documented spontaneous sustained VA but induced VA before ablation procedure. Notably, six patients (2%) had not had any documented spontaneous or inducible VA prior to their ablation procedure [12, 20, 23, 54]. A total of 43/91 patients (47%) had previously documented VF storm.

### Procedural characteristics

**Fig. 2:**
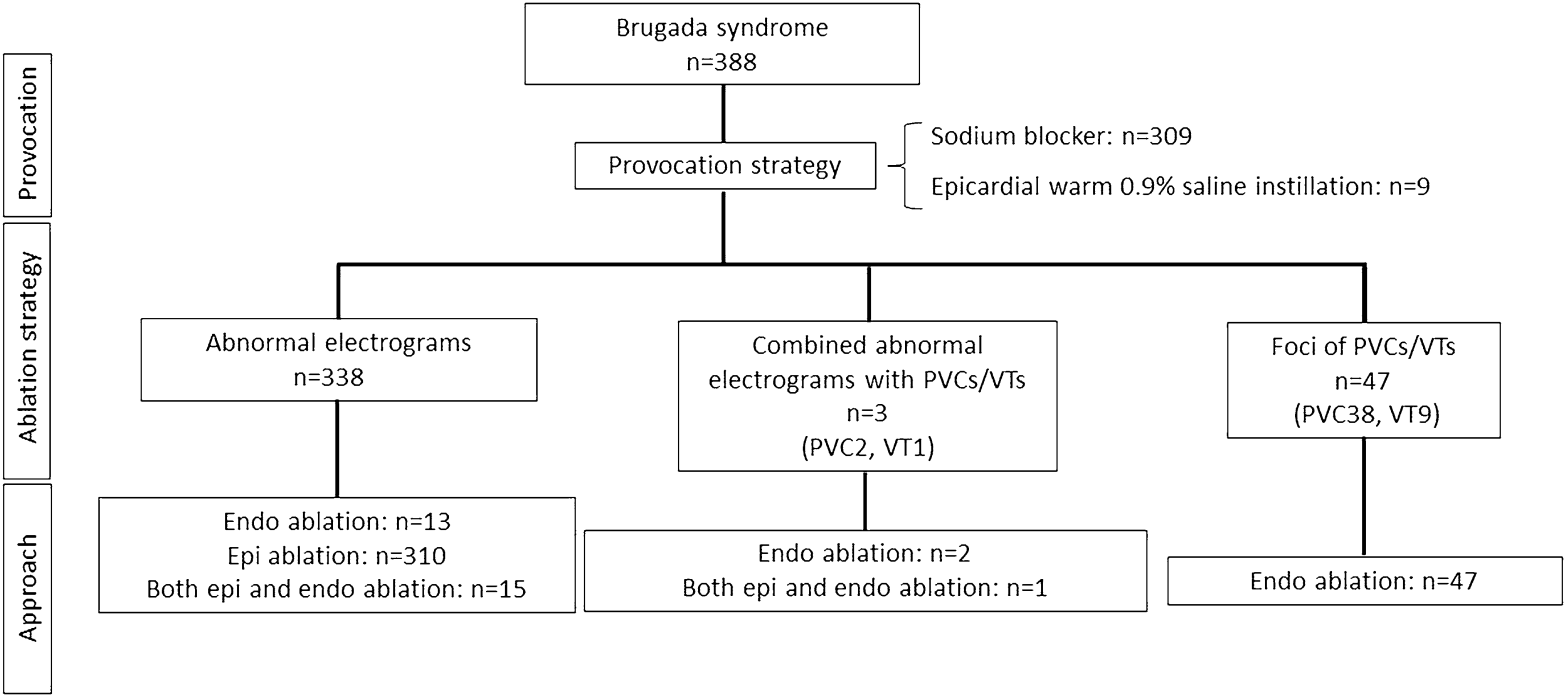
Ablation strategy and approach in patients with Brugada syndrome. *Endo* endocardium, *Epi* epicardium, *PVC* premature ventricular complex, *VT* ventricular tachycardia

The ablation strategy and approach in all 56 studies with 388 patients are summarized in Fig. 2. A variety of mapping was used. These included targeting of abnormal EGMs (defined as a combination of low voltage, late potentials or fractionated potentials) in 338 cases (87%), and targeting foci of PVCs or monomorphic VT in 47 cases (12%; PVC 38 cases, monomorphic VT 9 cases). An approach of combining targeting of abnormal EGMs and targeting PVC/VT was performed in three cases (1%).

### Provocation strategy

Following baseline electroanatomic mapping, pharmacological provocation was used to augment the arrhythmogenic substrate in 316/388 cases (81%). The provocation strategy was described in Table 2. The most frequently used strategy was sodium channel blocker in 309/316 cases (98%). Of these, ajmaline was the most frequently used in 235/309 cases (76%), following by pilsicainide in 36/309 cases (12%), flecainide in 20/309 cases (6%), propafenone in 10/309 cases (3%), and procainamide in 8/309 cases (3%). In nine cases, warmed 0.9% saline (39–40℃) was infused into the epicardial space [14, 55, 57, 58]. Two patients received both sodium channel blocker and epicardial warm water instillation [57, 58].

**Table 2:** Provocation strategy of Brugada phenotype

Provocation strategy of Brugada phenotype	
**Augmentation of arrhythmogenic substrate**	***316/388 (81%)***^***a***^
**Sodium channel blocker**	***n=309***
Ajmaline	235 (76%)
Pilsicainide	36 (12%)
Flecainide	20 (6%)
Propafenone	10 (3%)
Procainamide	8 (3%)
**Other strategy**	***n=9***
Epicardial warmed (39–40 ℃) 0.9% saline instillation	9 (100%)

^a^Two patients were used both sodium channel blocker and epicardial warm water instillation

### Location of abnormal substrate

**Fig. 3:**
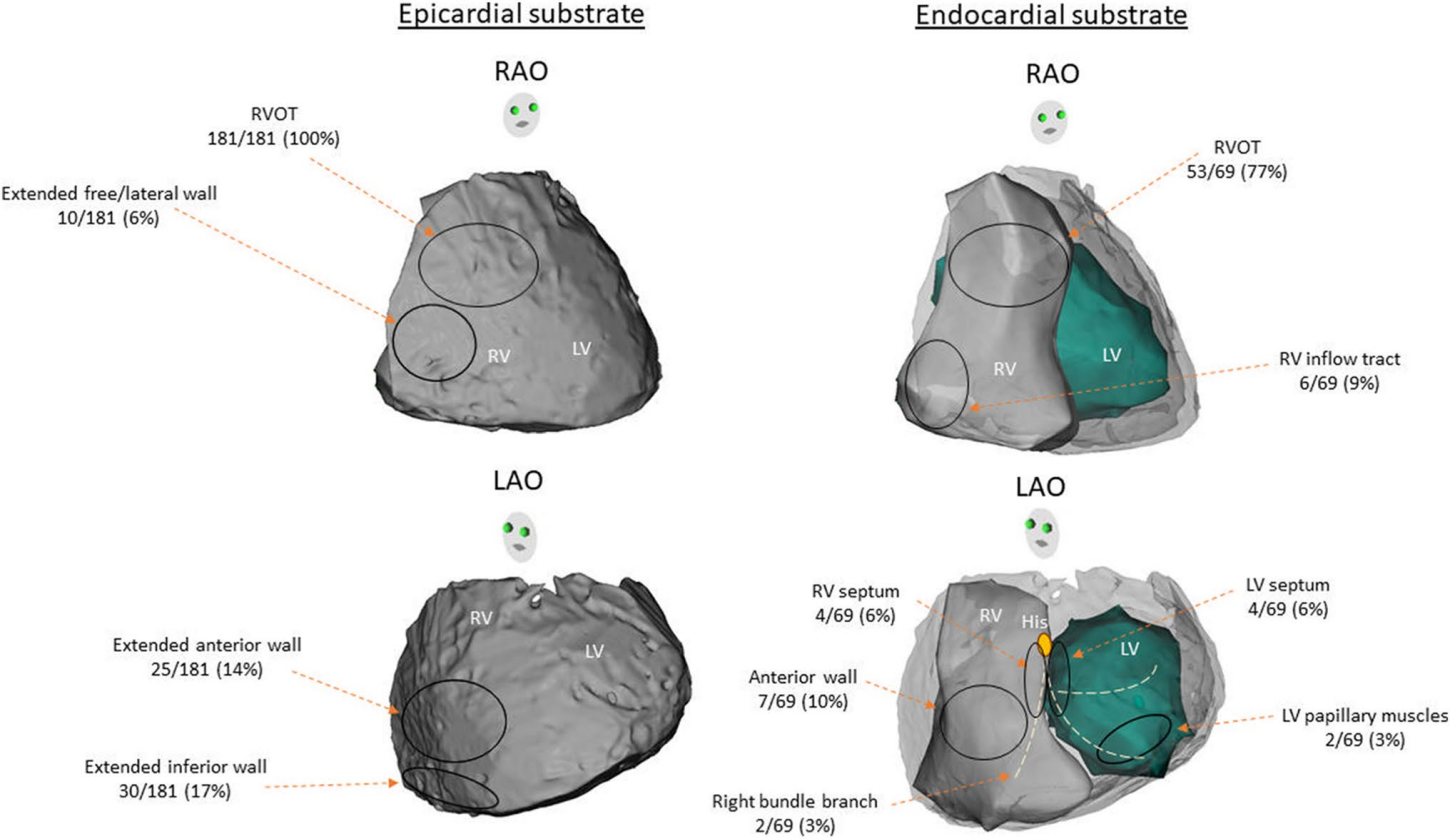
Localization of abnormal substrate**.** Pictorial description of localization of abnormal substrate showing that majority were from epicardial RVOT. In the majority of cases, abnormal substrate localized to the epicardial RVOT, however, extension of the abnormal substrate was highly variable, particularly after administration of sodium channel blocker. *LAO* left anterior oblique, *LV* left ventricular, *RAO* right anterior oblique, *RV* right ventricular, *RVOT* right ventricular outflow tract

Subsequent to pharmacological or thermal provocation, substrate mapping was performed. A summary of the location of arrhythmogenic substrate is described in Fig. 3. A total of 330 patients underwent epicardial mapping including four patients who were ablated from the endocardium alone. In the majority of cases, abnormal substrate localized to the epicardial RVOT, however, extension of the abnormal substrate was highly variable, particularly after provocation. Detailed substrate localization was not available in 149 epicardial cases [5, 9]. Of the remaining 181 cases where epicardial ablation was performed, all cases involved epicardial RVOT (100%) [3, 7, 8, 12–14, 16–18, 20–32, 48, 50, 51, 54–59], with extension to the RV free wall in 10 cases (6%) [7, 16, 25, 33, 54, 59], RV anterior wall in 25/181 cases (14%) [17, 27, 31, 54, 58] and RV inferior wall in 30 cases (17%) [17, 18, 26, 30, 31, 51, 52, 58, 59].

Data on endocardial substrate localization were available in all except for two of the 71 cases who underwent endocardial mapping (inclusive of those undergoing combined approach) [20, 32]. Of the remaining 69 cases, 53 cases (77%) involved the endocardial RVOT [7–11, 13–15, 18, 21, 34–41, 43, 44, 50, 53], followed by RV anterior wall in 7 (10%) [7, 10, 11, 53], RV inflow tract in 6 (9%) [11, 15, 18, 26, 43, 46], and RV septum in four (6%) [7, 19, 42].

**Fig. 4:**
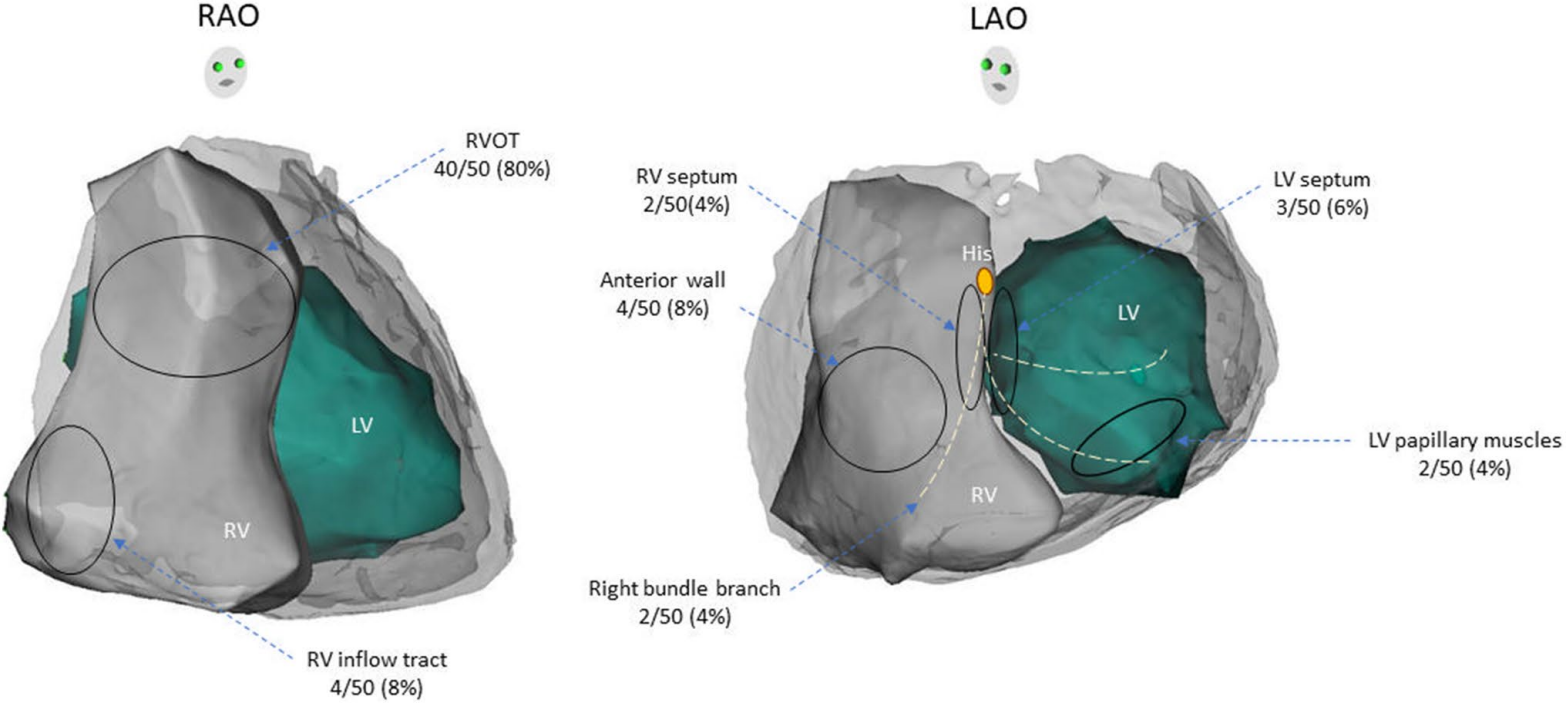
Origin of PVCs and VT foci. Pictorial description of PVC/VT foci showing that majority were from RVOT. All PVC-triggered VF and sustained VTs were localized to endocardium RVOT with highly variable involvement of multiple regions among both RV and LV endocardium. *LAO* left anterior oblique, *LV* left ventricular, *RAO* right anterior oblique, *RV* right ventricular, *RVOT* right ventricular outflow tract

Fifty out of 388 cases (13%) in patients with BrS were targeted for foci of PVCs and VTs including 47 cases targeted for PVC/VT foci alone and three cases were combined ablation strategies with abnormal substrate (Fig. 2). All PVCs and VTs were ablated from endocardium. PVCs and VTs were implicated in 40 and 10 patients, respectively. The majority of PVCs and VTs were localized to the endocardial RVOT. However, some of the PVCs and VTs were localized to other endocardial non-RVOT sites including four (8%) [11, 15, 43, 46] from RV inflow tract, four (8%) [10, 11, 53] from RV anterior wall, two (4%) [19, 42] from RV septum, two (4%) [15, 45] from conduction system of right bundle branch, three (6%) [15, 46, 48] from LV septum, and two (4%) [14] from LV papillary muscle (Fig. 4).

### Procedural endpoints

The primary endpoint of each study was described in Table 3. Resolution of type 1 ECG was the major primary endpoint in 143/388 cases (37%), followed by abolition of abnormal EGM in 132/388 (34%), combination of resolution of type 1 ECG with non-inducibility of VA in 39/388 cases (10%), non-inducibility of VA in 30/388 cases (8%).

**Table 3:** Primary endpoints

Primary endpoints	*n* = 388
Resolution of type 1 ECG	143 (37%)
Abolition of abnormal EGM	132 (34%)
Non-inducibility of VA	30 (8%)
Resolution of type 1 ECG + non-inducibility of VA	39 (10%)
Abolition of abnormal EGM + non-inducibility of VA	12 (3%)
Resolution of type 1 ECG + abolition of abnormal EGM	7 (2%)
Not specified	25 (6%)

*ECG*, electrocardiogram, *EGM*, electrogram, *VA*, ventricular arrhythmia

### Procedural outcomes

In majority of studies, follow-up data were acquired by repeated visits at the outpatient clinic. Each visit included 12-lead ECG, 24 h ambulatory ECG, and device-interrogation (supplemental table).

In 15 case series of 347 patients, the pooled incidence of non-inducibility of VA was achieved in 87.1% (95% CI 73.4–94.3; *I*^2^ = 51%), normalization of abnormal EGMs in 92.9% (95% CI [84.8–96.8]; *I*^2^ = 29%), and acute resolution of type I ECG in 74.5% (95% CI [52.3–88.6]; *I*^2^ = 75%) after ablation procedure. During a weighted mean follow-up of 28 months, 7.6% (95% CI [2.1–24]; *I*^2^ = 67%) had recurrence of type I ECG either spontaneously or with drug challenge [3, 6, 12, 13] as well as 17.6% (95% CI [10.2–28.6]; *I*^2^ = 60%) of sustained VA [3, 6–9, 11–18]. Similarly, in case studies of 41 patients, the endpoints of non-inducibility of VA, normalization of abnormal EGM and acute resolution of type I ECG occurred in 11/13 (85%), 20/21 (95%) and 16/21 (76%), respectively. During a median follow-up period of 16 (9–24) months, 2/16 (13%) had recurrence of type I ECG [38, 46] and 5/41 (12%) of sustained VA [32, 38, 42, 44, 46].

### Complications

Among 15 case series of 347 patients undergoing epicardial ablation, pericarditis or pericardial effusion occurred in 9.3% of patients (95% CI 5–16.6; *I*^2^ = 46%) [3, 5–8, 12, 17], and were self-limiting by conservative management in all cases. In total, two patients who refused ICD insertion died from sudden cardiac death. The first case [8] was epicardial approach targeting abnormal EGM in RVOT after sodium channel antagonist augmentation. Type I ECG had normalized at day 2 following the procedure, however, a recurrence of type II ECG was noted at 9 months without attempt at drug provocation to see whether type I ECG was produced. His prior VF burden was not documented.

The second case [46] was endocardial approach targeting PVCs in RV inflow tract and LV post-inferoseptum following administration of sodium channel antagonist. Initial resolution of type I ECG was achieved, however recurrence noted at 1 month post-procedure. He had documented episodes of VF during hospitalization in the months prior to his ablation, which was performed 4 months after his initial diagnosis of BrS due to refusal of ICD insertion.

## Discussion

A body of research spanning almost two decades describes the potential utility of catheter ablation as a therapeutic option for reduction or complete abolition of recurrent arrhythmic risk in BrS. This review summarizes localization of abnormal substrate, the ablation strategy, and clinical outcomes following catheter ablation in patients with BrS. This review provides several novel observations pertaining to catheter ablation for ventricular substrate in BrS. The main findings of this study are:

- 1. Ablation strategy

The vast majority of patients (87%) underwent catheter ablation based on the substrate modification, however, a significant minority of patients (13%) underwent mapping and ablation of PVC-triggered VF (10%) or ablation for sustained monomorphic VT (3%).

- 2. Provocation strategy

Eighty percent of patients received sodium channel blocker as a provocation strategy prior to catheter ablation to uncover the necessary “substrate” for ablation.

- 3. Localization of abnormal substrate and PVC/VT foci

Almost all abnormal substrates were localized in epicardial RVOT with possible extension. Beyond RVOT in 6% of cases to the RV free wall, 14% to RV anterior wall, and 17% to RV inferior wall. In contrast, all PVC-triggered VF and sustained VTs were localized to endocardium RVOT with highly variable involvement of multiple regions among both RV and LV endocardium.

- 4. Outcome of catheter ablation

Non-inducibility of VA was achieved in 87.1% and 17.6% had recurrence of sustained VA during approximately 2 years’ follow-up; complications occurred in 9.3% suggesting that catheter ablation is an excellent therapeutic option for treatment of VA associated with BrS.

Contemporary work has focused upon the epicardial RVOT as the region of interest in BrS, supported by the findings of epicardial RVOT fibrosis on autopsy in six patients [4]. However, in our study, we found that the abnormal substrate extended beyond the RVOT in approximately one third of patients for both endocardial and epicardial mapping. Some reported that many spontaneous episodes of VF in patients with BrS were preceded by PVCs [60]. In corresponding with these reports, 13% of patients underwent ablation of VF-triggering PVCs or VT ablation with or without substrate ablation.

Pharmacologic provocation with a sodium channel blocker was the cornerstone of identifying ablation targets. Salghetti, et al. reported that the substrate was visible in 17/36 (47%) of patients but required provocation for revelation in 19/36 (53%) of patients [12]. In almost all cases, provocation revealed a significant increase in abnormal substrate. Furthermore, the same pharmacological provocation could be used to test the efficacy of catheter ablation, where re-testing revealed abolishment of targeted substrate. An important point to note is that the definition of “abnormal substrate” encompassed both abnormal low bipolar voltage (< 1.5 mV) as well as late potential and fractionated potentials and low frequency (up to 100 Hz) prolonged duration (> 200 ms) bipolar signals with delayed activity extending beyond the end of QRS complex, which could be present in a low voltage area. These differing definitions of abnormal substrate are thematically consistent with the fiercely debated pathophysiologic mechanism of BrS, which is thought to be due to depolarization and/or repolarization abnormalities. It is considered that low bipolar voltage may reflect depolarization abnormalities and low-frequency, prolonged duration signals may reflect repolarization abnormalities. Although the underlying pathophysiology of BrS remains controversial, nevertheless, the provocation strategy is thought to be effective for both definitions of abnormal substrate as provocation showed a significant increase of abnormal substrate area not only low voltage area, but also slow conduction area in previous studies, with excellent procedural outcomes with targeting of substrate with catheter ablation. Ajmaline was the most frequently used for provocation drug in this pooled analysis. It is unclear which sodium channel blocker is the optimal drug, at the most appropriate dose. Further studies will be required to elucidate the optimal provocation strategy. A combined epicardial and endocardial approach might be required with sodium channel blocker augmentation, in addition to PVC and VT ablation where VF triggering PVCs and VTs have been identified.

Looking at the procedural outcomes, acute procedural endpoints of non-inducibility of VA were achieved in a large proportion of patients (87.1%) and long-term recurrence of sustained VA during median follow-up period of 28 months occurred in 17.6%. This data suggest reasonable success with the procedures. Complications occurred in 9.3% with the majority related to epicardial access including pericardial effusions or pericarditis, managed with a pericardial drain or medication, none requiring cardiac surgery. Death was observed in two patients who did not underwent ICD implantation out of 388, and unrelated to procedural complication. These results highlight the necessity for ICD in these patients given the risk of recurrent arrhythmia even if catheter ablation is apparently successful.

Considering highly variable distribution of abnormal substrate and ectopy with small proportion of SCN5A mutation suggest that BrS might be a heterogeneous entity because current definition of HRS/EHRA/APHRS expert consensus statement is based on ECG criteria alone. Theoretically, any disease that affects the epicardial RVOT myocardium can manifest with a coved‐type ECG and should be designated as Brugada phenocopy. The abnormal substrate, whilst initially localizing to epicardial RVOT, now appears to involve all aspects of the RV, suggesting that we need to start reconfirming the diagnosis, and look for alternative diagnosis, such as ARVC [61] or coronary ischemia (e.g., right coronary artery) which is also known to produce a coved‐type ST‐elevation and emulate BrS [62]. More careful phenotyping might be required to diagnose BrS as a unique entity. Nevertheless, contemporary catheter ablation of BrS should be the good adjunctive to ICD implantation to prevent recurrent VA.

One previous systematic review has described catheter ablation of BrS. Fernandes, et al. reported the efficacy of epicardial substrate modification in patients with BrS [63]. However, detailed localization of abnormal substrate and VF-triggered PVC/VT origin in BrS, and detailed comprehensive verification of clinical outcomes was still lacking. The efficacy of catheter ablation was reported to be favorable in this study similar to ours, but included only 11 case series and 11 case reports with 233 patients. Our study has several advantages, including an updated review, including a larger cohort of 388 patients, a comprehensive description of substrate location, in particular beyond epicardial RVOT and the reporting of procedural outcome using single-arm meta-analysis of case series and case reports. Uniquely, we included case series and case reports, to provide a more comprehensive representation of published literature on catheter ablation of BrS.

## Conclusion

Catheter ablation appears to be a safe and efficacious strategy for eliminating of VA and/or arrhythmogenic substrate for VA in BrS. Further study is needed to identify which patients stand to benefit, and optimal protocol for identifying ablation targets with pharmacological and/or thermal provocation.

### Supplementary Information

Below is the link to the electronic supplementary material.

Supplementary file1 (DOCX 18 KB)

Supplementary file2 (DOCX 15 KB)

## Data Availability

The data underlying this article will be shared on reasonable request to the corresponding author.

## Abbreviations

ARVC: Arrhythmogenic right ventricular cardiomyopathy
BrS: Brugada syndrome
CI: Confidence interval
ECG: Electrocardiogram
EGM: Electrogram
Endo: Endocardium
Epi: Epicardium
ICD: Implantable cardioverter defibrillator
IQR: Interquartile range
LV: Left ventricular
MRI: Magnetic resonance imaging
PVC: Premature ventricular complex
RV: Right ventricular
RVOT: Right ventricular outflow tract
VA: Ventricular arrhythmia
VF: Ventricular fibrillation
VT: Ventricular tachycardia
