# Correction to: Efficacy and safety of catheter ablation for Brugada syndrome: an updated systematic review

**DOI:** 10.1007/s00392-022-02038-7

**Published:** 2022-06-02

**Authors:** Yasuhito Kotake, Sumita Barua, Samia Kazi, Sohaib Virk, Ashwin Bhaskaran, Timothy Campbell, Richard G. Bennett, Saurabh Kumar

**Affiliations:** 1 Department of Cardiology, Westmead Hospital, Westmead, NSW Australia; 2 Westmead Applied Research Centre, University of Sydney, Westmead, NSW Australia

## Correction to: Clinical Research in Cardiology 10.1007/s00392-022-02020-3

In Table 1, the value for the 'Initial presentation' should have read n = 342. Additionally, in Table 2, the value for the 'Augmentation of arrhythmogenic substrate' should have read 316/388 (81%)^a^.

The original article has been corrected.

