# Supplementary material for: Efficacy and safety of catheter ablation for Brugada syndrome: an updated systematic review
Source: Clin Res Cardiol. 2022 Apr 22;112(12):1715–26. doi: 10.1007/s00392-022-02020-3 (PMC10698106; doi:10.1007/s00392-022-02020-3)
Supplement: Supplementary file 1 — Supplementary file1 (DOCX 18 KB) [file 392_2022_2020_MOESM1_ESM.docx]

**Supplemental methods**

**Study eligibility**

Exclusion criteria were review articles, editorials, letters, conference presentations and studies that did not meet the inclusion criteria described on the manuscript. Additional studies were included by manual searching of reference lists in review articles on BrS. Study eligibility was independently determined by 3 investigators (S.B., S. Kazi, Y.K.). All 3 investigators screened titles and abstracts of all relevant studies which were systematically searched, and manually searched the full-text version of all studies that fulfilled the inclusion criteria. Any differences were resolved by consensus between authors following complete review of the article. Quality assessment was performed objectively using the Joanna Briggs Institute Checklist for Case Reports and Case Series.

**Data Extraction**

A standardized data collection form was used to obtain the following information from each study; title of study, name of first author, number of patients, demographic data of participants (age, gender, initial presentation, previous ventricular arrhythmia, presence of SCN5A mutation, spontaneous type I electrocardiogram [ECG] and prior VF storm). Procedural characteristics collected including ablation strategy, localization of abnormal substrate and primary endpoints. Outcome data collected including incidence of non-inducibility of ventricular arrhythmia (VA), acute resolution of type 1 ECG, acute and long-term complication (pericarditis, death) and recurrence of type I ECG pattern or VA. This data extraction process was independently performed to ensure accuracy. Discordant data was resolved by referring back to each article and consensus between authors.
