# Supplementary material for: Efficacy and safety of catheter ablation for Brugada syndrome: an updated systematic review
Source: Clin Res Cardiol. 2022 Apr 22;112(12):1715–26. doi: 10.1007/s00392-022-02020-3 (PMC10698106; doi:10.1007/s00392-022-02020-3)
Supplement: Supplementary file 2 — Supplementary file2 (DOCX 15 KB) [file 392_2022_2020_MOESM2_ESM.docx]

**Supplemental table. Follow-up details for all case series**

| Study (Year) | Sample size | Follow up (months) | Holter-monitoring | Device-interrogation | Outpatient clinic |
| --- | --- | --- | --- | --- | --- |
| Nademanee, et al. 2011^3^ | 9 | 20 | NA | NA | 1 month and every 3 months |
| Brugada, et al. 2015^5^ | 14 | 5 | At each follow-up visits | NA | NA |
| Pappone, et al. 2017 ^6^ | 135 | 10 | 3 month, 6 month, 12 month and every 6 months | 3 month, 6 month, 12 month and every 6 months | 3 month, 6 month, 12 month and every 6 months |
| Shelke, et al. 2018 ^7^ | 5 | 46 | NA | NA | 1 week, 2 week, 3 week, 3 months and every 6 months |
| Zhang, et al. 2016 ^8^ | 11 | 31 | NA | NA | 1 month and every 3 months |
| Sunsaneewita, et al. 2012 ^9^ | 4 | 26 | NA | 1 month and every 3 months | 1 month and every 3 months |
| Haissaguerre, et al. 2003 ^10^ | 3 | 17 | 1 month | NA | NA |
| Talib, et al. 2018 ^11^ | 21 | 55 | NA | NA | NA |
| Salghetti, et al. 2019 ^12^ | 36 | 16 | 1 month and every 6 months | 1 month and every 6 months | 1 month and every 6 months |
| Haanshoten, et al. 2020 ^13^ | 6 | 43 | NA | NA | NA |
| Chung, et al. 2017 ^14^ | 15 | 18 | Every 3 to 6 months | NA | Every 3 to 6 months) |
| Tokioka, et al. 2020 ^15^ | 7 | 86 | NA | Every 3 months | Every 3 months |
| Mamiya, et al. 2021 ^16^ | 11 | 42 | NA | Every 6 months | 1, 3, 6, and every 6 months |
| Chokesuwattanaskul R, et al. 2021 ^17^ | 54 | 32 | NA | NA | NA |
| Kamakura, et al. 2021 ^18^ | 16 | 25 | NA | Every 6 to 12 months | Every 6 to 12 months |

**Abbreviations**: NA, not available
